# A cost-utility analysis of nursing intervention via telephone follow-up for injured road users

**DOI:** 10.1186/1472-6963-9-98

**Published:** 2009-06-11

**Authors:** Carin Franzén, Ulf Björnstig, Christine Brulin, Lars Lindholm

**Affiliations:** 1 Department of Nursing, Division of Surgery, Umeå University, S-901 87 Umeå, Sweden; 2 Department of Surgical and Perioperative Sciences, Division of Surgery, Umeå University, S-901 87 Umeå, Sweden; 3 Department of Surgical and Perioperative Sciences, Division of Surgery, Umeå University, S-901 87 Umeå, Sweden; 4 Department of Nursing, Umeå University, SE-901 87 Umeå, Sweden; 5 Department of Public Health and Clinical Medicine, Umeå University, 901 87 Umeå, Sweden

## Abstract

**Background:**

Traffic injuries can cause physical, psychological, and economical impairment, and affected individuals may also experience shortcomings in their post-accident care and treatment. In an earlier randomised controlled study of nursing intervention via telephone follow-up, self-ratings of health-related quality of life were generally higher in the intervention group than in the control group.

**Objective:**

To evaluate the cost-effectiveness of nursing intervention via telephone follow-up by examining costs and quality-adjusted life years (QALYs).

**Methods:**

A randomised controlled study was conducted between April 2003 and April 2005. Car occupants, cyclists, and pedestrians aged between 18 and 70 years and attending the Emergency Department of Umeå University Hospital in Sweden after an injury event in the traffic environment were randomly assigned to an intervention (n = 288) or control group (n = 280). The intervention group received routine care supplemented by nursing via telephone follow-up during half a year, while the control group received routine care only. Data were collected from a mail survey using the non-disease-specific health-related quality of life instrument EQ5D, and a cost-effectiveness analysis was performed including the costs of the intervention and the QALYs gained.

**Results:**

Overall, the intervention group gained 2.60 QALYs (260 individuals with an average gain of 0.01 QALYs). The car occupants gained 1.54 QALYs (76 individuals, average of 0.02). Thus, the cost per QALY gained was 16 000 Swedish Crown (SEK) overall and 8 500 SEK for car occupants.

**Conclusion:**

Nursing intervention by telephone follow-up after an injury event, is a cost effective method giving improved QALY to a very low cost, especially for those with minor injuries.

**Trial registration:**

This trial registration number is: ISRCTN11746866.

## Background

Road traffic injuries are a major global problem. The World Health Organisation (WHO) currently ranks traffic crashes as the ninth leading cause of disability, and it has been estimated that by 2020 they will have risen to third place, behind heart disease and depression [1]. In Sweden, 1.4 per 100 inhabitants are injured in the traffic environment each year [2]; the three most frequently injured categories are car occupants, cyclists, and pedestrians, injured for example by falls (Swedish Institute for Transport and Communication Analysis, SIKA 2006:31). The economic cost of road crashes and injuries is enormous. The global cost is conservatively estimated to be between 1% and 2% of gross national product [3]. In addition, earlier studies have also shown QALY losses after injury events in the traffic environment [4,5]. In a study of the value of a statistical life in the road traffic sector, Persson et al. [6] estimated the value of a statistical life in 1999 prices at 20 million Swedish crown (SEK), that of a severe casualty at 3.3 million SEK, and that of a minor casualty at about 0.3 million SEK.

Since traffic injuries are an increasing global problem, it is important not only to prevent injuries but also to provide proper care and treatment to the injured persons. Earlier studies have indicated shortcomings in treatment, care and rehabilitation after an injury event. For example, Franzén et al. [7] pointed out that persons with non-minor injuries rated their quality of care in the emergency department after injury event higher than those with minor injuries, even if the importance for quality of care was the same for both groups. Furthermore, Cedergren & Bylund [8] and Albertsson & Björnstig [9] showed that injured people lacked both support and information from their caregivers during a hospital stay, and were also uncertain of where to go for help with any further needs after discharge. Moreover, Franzén et al. [10] showed that lack of information from caregivers created perceived feelings of anxiety and uncertainty among the injured. In addition, several studies has indicated that an awareness of those injured persons who need more extensive support is important for both quality of care and health-related quality of life [11-13]. A number of authors have also pointed out the need for more effective interventions after an injury event [14-17]. Nursing intervention by telephone follow-up (TFU) could offer one way to provide such intervention.

In an earlier randomised controlled intervention study [18] we investigated three road user categories (car occupants, cyclists, and pedestrians) who attended the Emergency Department of Umeå University Hospital in northern Sweden. Both groups received routine care according to current standard trauma principles, ATLS [19] and TNCC [20], and the intervention group also received nursing intervention by TFU after discharge from the hospital. The intervention calls lasted an average of 20 minutes (SD = 9.56), and 62% needed specific intervention advice. Patients' concerns were classified into six major areas and ranked according to frequency. The six areas were: self-care (29%), recommendation to seek further medical attention at the local hospital's medical centre (26%), explanation of symptoms (25%), recommendation to seek a physiotherapist (11%), information on prognosis (5%), and pharmacological information (4%). The main results from the study showed that, in general, the intervention group rated their health-related quality of life significantly higher than did the control group. This improvement was most pronounced in the group of those provided with advice as part of TFU. The car occupants gained most advantage from the TFU, with significantly lower problems in the dimensions of pain/discomfort and usual activities.

Earlier economic studies including TFU as a part of the intervention have been conducted for various patient categories, mainly in the context of cardiac rehabilitation [21-23], antibiotic prescriptions [24], early obstetrical discharge [25], depression [26], telephone triage in general practice [27], telephone triage for asthma [28], telephone triage for patients in the National Health Service [29], and advice on newly prescribed medicines [30]. These studies showed that while these interventions are generally less costly, except in a couple of cases [29,30] they are also ineffective. However, our TFU study showed significant higher QALY scores in the intervention group than in the control group (Table 1). In an environment where health care resources are scarce, it is important not only to show that a follow-up method is effective, but also to demonstrate that it offers value for money. Nevertheless, to our knowledge, the cost-utility of nursing intervention via TFU after an injury event in the traffic environment has not yet been clarified.

**Table 1:** Mean of EQ-5D index for the responders at baseline in the intervention group (n = 288) and control group (n = 280) and after 6 months in the intervention group (n = 260) and control group (n = 250).

**EQ-5D Index**			
	**EQ-5D** **Baseline**	**EQ-5D** **3 months**	**EQ-5D** **6 months**

**Groups**	**Mean (SD)**	**Mean (SD)**	**Mean (SD) Confidence interval (CI)**

Intervention group	0.64 (0.31)	0.80 (0.22)	0.85 (0.20) 0.8209–0.8698

Control group	0.64 (0.31)		0.81 (0.21) 0.7842–0.8359

**P-value**	**0.92**		**0.05**

Intervention car occupants	0.71 (0.28)	0.81 (0.23)	0.87 (0.18) 0.8323–0.9141

Control car occupants	0.68 0.32)		0.79 (0.21) 0.7406–0.8402

**P-value**	**0.44**		**0.01**

Intervention cyclists	0.66 (0.30)	0.84 (0.21)	0.86 (0.21)

Control cyclists	0.68 (0.28)		0.86 (0.17)

**P-value**	**0.60**		**0.92**

Intervention pedestrians	0.55 (0.32)	0.75 (0.20)	0.81 (0.20)

Control pedestrians	0.57 (0.32)		0.78 (0.24)

**P-value**	**0.72**		**0.29**

The aim of the present study was to evaluate the cost-effectiveness of nursing intervention via telephone follow-up by examining costs and quality-adjusted life years.

## Methods

### Selection procedure

A randomised controlled trial design was used. Data were collected from April 2003 to April 2005. Firstly, a stratified consecutive sample procedure was used to select a representative sample of participants from three different road user categories: car occupants, cyclists, and pedestrians (falls, non-vehicle injuries). Secondly, a randomised procedure was used to allocate each patient to either the intervention or the control group. The sample size was determined by power analysis based on a significance level of 0.05 and power = 0.90. A total of 300 participants in each group were necessary to find a difference of 0.03 in the EQ-5D index, and similarly for subgroups of size 150 participants in each group a difference of 0.05 were adequate.

**Figure 1:**
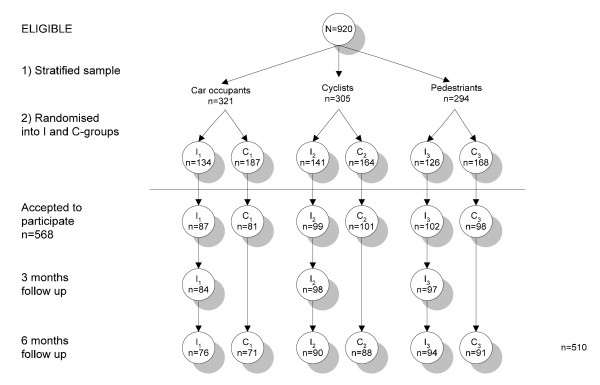
**Selection and data collection procedure for the intervention and control groups**. *I = Intervention group. *C = Control group.

Detailed descriptions of the selection procedure for the intervention and the control group and the data collection procedure are given in Figure 1.

### Participants

The inclusion criteria in this study were: (1) age between 18 and 70 years; (2) being; (i) car occupant, (ii) cyclist, or (iii) pedestrian; and (3) having attended the Emergency Department of Umeå University Hospital in northern Sweden after an injury event in the traffic environment. Participants were excluded if they were diagnosed with dementia or mental illness. The injuries were classified according to the Abbreviated Injury Scale (AIS), where MAIS denotes the Maximum AIS. AIS = 1 is a minor injury (e.g. whiplash injury, sprain, finger fracture), AIS = 2 is a moderate injury (e.g. concussion, radius fracture), AIS = 3 is a serious injury (femoral fracture, intra-abdominal bleeding), and the scale continues up to AIS = 6, which covers fatal injuries [31]

In our results, we present two injury groups; those with minor injuries (MAIS = 1) and those with moderate and more serious injuries (MAIS = 2+). Following the power calculation, a total of 920 individuals were invited to participate; 568 of them accepted the invitation. Background characteristics for the responders in the intervention group and control group are shown in Table 2. Participants and refusals differed in that there were more males among the refusals (59%) than among the participants (38%), and there were also more MAIS 1 injuries among the refusals (77% vs. 63%). The refusals were also slightly younger, with a mean age of 36.8 (SD: 13.6) as compared to 43.8 (SD: 15.1) years.

**Table 2:** Background characteristics for the responders in the intervention group (n = 288) and the control group (n = 280).

	**Car occupants (n = 87)**	**Car occupants** **(n = 81)**	**P-value**	**Cyclists** **(n = 99)**	**Cyclists** **(n = 101)**	**P-value**	**Pedestrians** **(n = 102)**	**Pedestrians** **(n = 98)**	**P-value**	**Total** **(n = 288)**	**Total** **(n = 280)**	**P-value**
	**Intervention**	**Control**		**Intervention**	**Control**		**Intervention**	**Control**		**Intervention**	**Control**	

**Age**	38.47 (12.02)	38.63 (13.98)	**0.938**	42.28 (15.33)	39.71 (15.38)	**0.238**	51.00 (13.00)	51.29 (14.52)	**0.880**	44.22 (14.51)	43.25 (15.73)	**0.547**

**MAIS**												

**1**	79 (91%)	73 (90%)	**0.580**	58 (59%)	58 (57%)	**0.984**	47 (46%)	43 (44%)	**0.552**	184 (64%)	174 (62%)	**0.823**
							
**2+**	8 (9%)	8 (10%)		41 (41%)	43 (43%)		55 (54%)	55 (56%)		104(36%)	106 (38%)	

**Sex**												

**Male**	47 (54%)	35 (43%)	**0.211**	33 (33%)	49 (49%)	**0.029**	26 (25%)	26 (27%)	**0.964**	106 (37%)	110 (39%)	**0.533**
							
**Female**	40 (46%)	46 (57%)		66 (67%)	52 (51%)		76 (75%)	72 (73%)		182 (63%)	170 (61%)	

**Marital status**												

**Not single**	72 (83%)	65 (80%)	**0.142**	68 (70%)	70 (69%)	**0.753**	87 (85%)	67 (69%)	**0.062**	228 (79%)	202 (72%)	**0.094**
							
**Single**	15 (17%)	16 (20%)		31 (30%)	31 (31%)		15 (15%)	31 (32%)		60 (21%)	78 (28%)	

**Education level**												

**Less than high school**	9 (10%)	11 (14%)		15 (15%)	13 (13%)		37 (36%)	30 (31%)		61 (21%)	54 (20%)	

**High school**	44 (51%)	42 (52%)	**0.696**	38 (38%)	36 (36%)	**0.762**	33 (33%)	37 (37%)	**0.686**	115 (40%)	115 (41%)	**0.884**
							
**University**	34 (39%)	28 (34%)		46 (47%)	52 (51%)		32 (31%)	31 (32%)		112 (39%)	111 (39%)	

**Work**												

**Employment**	66 (76%)	65 (81%)	**0.294**	62 (63%)	69 (68%)	**0.450**	74 (72%)	62% (64%)	**0.259**	202 (70%)	196 (70%)	**0.108**
							
**Retired to some extent**	9 (10%)	6 (7%)		12 (12%)	8 (8%)		21 (21%)	25 (25%)		42 (15%)	(39 (14%)	
							
**Student**	8 (9%)	6 (7%)		20 (20%)	21 (21%)		3 (3%)	10 (10%)		31 (11%)	37 (13%)	
							
**Unemployed**	4 (5%)	4 (5%)		5 (5%)	3 (3%)		4 (4%)	1 (1%)		13 (4%)	8 (3%)	

### Procedure

A letter with information about the study and an invitation to participate was sent to the invited participants in both the intervention and the control group about three weeks after the injury event. Informed consent was given written from the participants. In order to evaluate the intervention, the EQ-5D questionnaire was sent twice, first with the invitation letter and again after six months. The intervention group also received a questionnaire after three months.

EQ-5D is a non-disease-specific self-report instrument for measuring health-related quality of life. It consists of the EQ-5D self-classifier and the EQ visual analogue scale [32]. Respondents are asked to classify their own health status in five dimensions: mobility, self-care, usual activities, pain/discomfort, and anxiety/depression. Answers are given on a three point scale: 1 = no problems, 2 = moderate problems, and 3 = severe problems. Theoretically, 243 health statuses could be generated by this classification. Each health status can be given a value from -0.59 to 1.0 by means of the time-trade method (Dolan 1997).

For the economic analysis in the present study, the EQ-5D index values were used to calculate health gains in QALYs [33,34]. The QALY is an approximation of utility; it combines the time spent in a health state with the quality experienced during that time [35,36].

All costs presented are mean costs evaluated in 2008 SEK (Table 3). Health care costs include the cost of nursing time, which was based on mean wages for registered nurses. We have used the market decided wages for nurses, and we got the information from the administration of the Västerbotten county council in Sweden. The meanwage was 33 778 SEK per month and 211 SEK per hour. The clinic used in the trial is situated at the university, so telephone costs and overhead costs were obtained from the university department. The time spent on each telephone call was recorded, as was any time needed for preparation (reading medical records) and supplementary work (making notes after the telephone call).

**Table 3:** Calculations of times and costs for the total intervention group and for the car occupants.

**Time**				
**Telephone calls including before and after work**	**Intervention group (n = 288)**	**Intervention car occupants** **(n = 87)**	**Intervention cyclists (n = 99)**	**Intervention pedestrians** **(n = 102)**

**Total time**	180 hours (4.5 weeks)	59 hours (1.4 weeks)	58 hours (1.5 weeks)	66 hours (1.6 weeks)

**Costs for the total time**				

**Salary for the nurses**	38 000 SEK	12 000 SEK	-	-

**Rent for premises**	4 500 SEK	1 000 SEK	-	-

				

**Total**	42 500 SEK	13 000 SEK	-	**-**

**Mean cost per participant**	146 71 SEK	133 25 SEK	-	-
**(SD)**	42.21	33.97		
**Confidence interval (CI)**	141.82–151.61	128.53–137.98		

### Statistical analysis

The intervention and control group and their subgroups were compared with respect to the following background characteristics: MAIS, sex, marital status, education, and employment. *Chi2 *test was used and for age independent *t-test*. The outcome variable was self-rated health expressed as mean QALY score on the UK EQ-5D index tariff [37] and was analysed with independent *t-test*. All analysis was performed using SPSS version 13, with significance level set at p ≤ 0.05.

### Economic analysis

We conducted a cost-utility analysis from a health care perspective, and compared the incremental cost and incremental utility gain of TFU in the intervention group compared to no TFU in the control group. The analysis relied on the QALYs gained over the six-month period derived from the social tariff [38].

### Ethics

The study was approved by the Ethics Committee, Umeå University, Sweden (dnr 03-079).

## Result

### Quality-adjusted life years

At baseline, there were no significant differences in QALY score between intervention and control group. The intervention group also received a questionnaire after three months because we wanted to understand the lead period for the potential effect. We couldn't see the same reason for a 3 months measurement in the control group. Furthermore, even a questionnaire is a kind "light" intervention so they were excluded from the 3 month follow up. (Table 1). After six months, the QALY scores for intervention and control groups were 0.85 and 0.81 respectively (p = 0.05), with the greatest difference being seen among car occupants, who had scores of 0.87 and 0.79 respectively (p = 0.01). If we assume that the difference in QALY scores developed linearly, the gain per person was 0.01 QALYs for the intervention group as a whole (0.04/2 multiplied by 0.5 years) and 0.02 QALYs for car occupants (0.08/2 multiplied by 0.5 years). In total, the intervention group gained 2.60 QALYs (0.01 each for 260 individuals). The car occupants gained 1.54 QALYs (0.02 each for 76 individuals). The total costs for the intervention group were 42 500 SEK, and the intervention costs for the car occupant group were 13 000 SEK. Thus the cost per QALY was 16 000 overall and 8 500 SEK for car occupants.

We undertook a simple sensitivity analysis based on the confidence intervals for costs and effects for the intervention group. For the entire intervention group, the ratio was between 9 454 to 60 644 SEK per QALY gained. In the car occupants group, the ratio was between 4 284 and 13 798 SEK per QALY gained.

## Discussion and conclusion

This study shows that nursing intervention via telephone follow up is cost effective when used alongside standard care in patients who were injured in the traffic environment, and that the costs per gained QALY are low; 16 000 SEK overall and 8 500 SEK among car occupants. The calculations presented show that the cost per QALY for nursing intervention via telephone follow-up is considerably lower than the corresponding cost per QALY for the guiding principles from Swedish National Board of Health and Welfare, where low costs are defined as < 100 000 SEK per QALY, moderate costs as ≤ 500 000 SEK per QALY [39].

The strengths of this trial are the randomised controlled design and the large population. Moreover, the initial stratified sampling procedure made it possible to involve different types of road users, thus providing a good representation of the most injured road users in Sweden.

The time spent on each telephone call from the patient's perspective (for example, employment time) was not included in the calculations; however, most calls took place when the participants were at home. All direct costs caused by the nursing intervention via telephone follow-up were included, for example salary and rent for the premises. Because the evaluation focused on a narrow perspective, it was not able to evaluate the wider societal impact of the nurse intervention e.g. the impact on reducing work loss and productivity costs.

Although road traffic injuries constitute a major public health problem and impose large costs on society, economic evaluations concerning people injured in traffic incidents are scarce. The most similar patient groups represented in the literature are people with different kinds of neck complaints; the results of implementation studies are mixed. For example, Rosenfeld *et al*. [40], who studied people with whiplash injuries after traffic incidents, pointed out that the costs of interventions including information, postural control, and exercises were significantly lower after 6 and 36 months when compared with standard intervention, and the intervention group also showed significantly lower pain and less sick leave. On the other hand, Rebbeck *et al*. [41], who evaluated two implementation strategies for whiplash injury guidelines in physiotherapy, found that although the active implementation program increased guideline-consistent practice among the physiotherapists, the patient outcomes and cost of care were not affected. In a randomised trial, Lewis *et al*. [42] compared three different physiotherapy treatments for persons with non-specific neck disorders: advice and exercise plus manual therapy, advice and exercise plus pulsed shortwave diathermy, and advice and exercise alone. After six months, advice and exercise alone was the most cost-effective method and also that with the highest QALY scores. In summary, it seems that post-injury interventions which include advice provision are the most successful. In accordance with our results, advice seems to be a cost-effective method. One possible explanation for our results could be the dominance of minor injuries (MAIS 1) in the intervention group. Essentially, while in Sweden a follow-up visit will be booked for in principally all MAIS 2 injuries, such as fractures or other serious injures. This is not a routine for minor MAIS 1 injuries such as sprains or whiplash injuries.

Our results support that nursing intervention by telephone follow-up after an injury event is a very cost effective strategy giving an improved QALY to low cost, especially for those with minor injuries. Based on these results and noting that traffic injuries cause many subsequent problems for the injured parties, we recommend that health care providers consider implementing a nursing intervention program by telephone follow-up for all persons injured in the traffic environment.

## Competing interests

The authors declare that they have no competing interests.

## Authors' contributions

Study design: CF, LL; data collection: CF; data analysis: CF, UB, CB, LL; manuscript preparation: CF, UB, CB, LL.

## Pre-publication history

The pre-publication history for this paper can be accessed here:


